# Controlling DNA-end resection: a new task for CDKs

**DOI:** 10.3389/fgene.2013.00099

**Published:** 2013-06-03

**Authors:** Lorenza P. Ferretti, Lorenzo Lafranchi, Alessandro A. Sartori

**Affiliations:** Institute of Molecular Cancer Research, Faculty of Medicine, University of ZurichZurich, Switzerland

**Keywords:** DNA double-strand break repair, DNA-end resection, homologous recombination, cyclin-dependent kinase, phosphorylation, CtIP/Sae2, PIN1

## Abstract

DNA double-strand breaks (DSBs) are repaired by two major pathways: homologous recombination (HR) and non-homologous end-joining (NHEJ). The choice between HR and NHEJ is highly regulated during the cell cycle. DNA-end resection, an evolutionarily conserved process that generates long stretches of single-stranded DNA, plays a critical role in pathway choice, as it commits cells to HR, while, at the same time, suppressing NHEJ. As erroneous DSB repair is a major source of genomic instability-driven tumorigenesis, DNA-end resection factors, and in particular their regulation by post-translational modifications, have become the subject of extensive research over the past few years. Recent work has implicated phosphorylation at S/T-P motifs by cyclin-dependent kinases (CDKs) as a major regulatory mechanism of DSB repair. Intriguingly, CDK activity was found to be critically important for the coordinated and timely execution of DNA-end resection, and key players in this process were subsequently identified as CDK substrates. In this mini review, we provide an overview of the current understanding of how the DNA-end resection machinery in yeast and human cells is controlled by CDK-mediated phosphorylation.

## Introduction

In order to preserve genome integrity, cells employ a complex surveillance network that detects, signals and repairs DNA lesions. These intricate and highly regulated pathways are collectively termed the DNA damage response (DDR; Zhou and Elledge, 2000). One major hallmark of the DDR represents the activation of checkpoints to temporarily delay cell cycle progression through inhibition of cyclin-dependent kinase (CDK) activity. In the budding yeast *Saccharomyces cerevisiae*, a single CDK, Cdc28 (or Cdk1), drives both G1/S and G2/M transitions, whereas in metazoan four CDKs are responsible for cell cycle progression (Morgan, 1997). CDK activity is modulated by association with regulatory subunits known as cyclins, the levels of which oscillate during the cell cycle (King et al., 1996). G1 phase is controlled by CDK4 and CDK6 in complex with D-type cyclins, whereas CDK2-cyclin E is essential for G1/S transition and the assembly of the DNA replication machinery. CDK2-cyclin A is required for proper completion of DNA replication and progression through S phase. Toward the end of interphase, cyclin A associates with CDK1 to facilitate S/G2 transition before CDK1-cyclin B complexes drive cells through mitosis (Morgan, 1997; Malumbres and Barbacid, 2005). CDKs belong to a large family of proline-directed kinases (which also includes MAPKs and GSK3) that exclusively phosphorylate serines or threonines immediately preceding a proline (S/T-P motifs) (Hanks and Hunter, 1995; Errico et al., 2010). CDK substrate specificity is increased by direct binding of the cyclin subunit to conserved RxL motifs present in certain CDK targets (Harper and Adams, 2001). A recent study showed that 50% of CDK2-cyclin A targets carried at least one RxL motif distal to the phosphorylation site (Chi et al., 2008).

**Figure 1:**
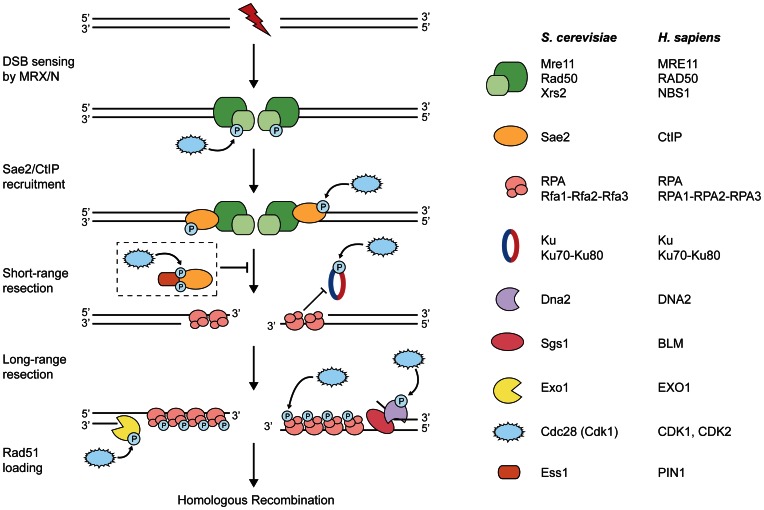
**CDKs target components of the DNA-end resection machinery**. Upon induction of a DNA double-strand break (DSB), the MRX/N complex rapidly localizes to the damaged site. During S and G2 phases of the cell cycle, DSB repair via homologous recombination (HR) is initiated by DNA-end resection. At first, short-range resection is carried out by the MRX/N complex and Sae2/CtIP; the two factors collaborate in the initial end trimming creating short 3′-ssDNA overhangs, which are immediately coated by replication protein A (RPA). Importantly, processed DSB ends are no longer suitable substrates for Ku binding and, thus, for the repair by non-homologous end-joining. Next, long-range resection is catalyzed either by the 5′-3′ exonuclease Exo1 or the helicase Sgs1/BLM in conjunction with the endonuclease Dna2. Subsequently, RPA is removed from ssDNA and replaced by the Rad51 recombinase that is required for strand invasion of the sister chromatid and further downstream steps in HR (not depicted in the figure). The dashed box depicts the proposed inhibitory role of PIN1 during DNA-end resection: PIN1 binds and subsequently isomerizes phosphorylated CtIP, thereby promoting its degradation by the proteasome and, hence, counteracting resection and HR. Note that both short- and long-range resection factors are potentially regulated by CDK phosphorylation (please refer to the main text for details).

In accordance with reduced CDK activity as a consequence of DNA damage-induced checkpoint activation, S/T-P motifs are largely dephosphorylated in response to DNA double-strand breaks (DSBs) (Bennetzen et al., 2010; Beli et al., 2012). However, in apparent contrast to this, CDK activity is strictly required for accurate processing and repair of DSBs in S/G2 phase, indicating that at least some DDR factors are primed by CDK phosphorylation prior to checkpoint activation (Enserink and Kolodner, 2010; Chapman et al., 2012). DSBs are highly deleterious lesions with the potential to cause cell death or genomic instability leading to cancer. DSBs can arise spontaneously as a result of replication fork collapse or can be induced by exogenous DNA-damaging agents including ionizing radiation and certain anti-cancer drugs (Jackson and Bartek, 2009). In order to repair DSBs, all organisms rely on two major pathways: non-homologous end-joining (NHEJ) and homologous recombination (HR). NHEJ functions throughout the cell cycle and religates broken ends without the need of extensive processing (Lieber, 2010). HR, instead, requires an undamaged template for faithful DSB repair, usually the sister chromatid, and is therefore restricted to S/G2 phase (Heyer et al., 2010). HR is initiated by 5′-3′ degradation of the DSB ends to generate 3′-single-stranded DNA (ssDNA) overhangs. This evolutionarily conserved process, termed DNA-end resection, requires the coordinated action of several nucleases and helicases (Figure 1; Mimitou and Symington, 2009; Blackwood et al., 2013). Recent work in yeast and human cells has established that DNA recombination and particularly DNA-end resection are highly regulated by various kinases including Mec1/ATR, Tel1/ATM, Rad53/CHK1, Cdc5/PLK1, and, as reviewed here, CDKs (Longhese et al., 2010; Chapman et al., 2012; Finn et al., 2012; Krejci et al., 2012).

## CDK substrates in DNA-end resection

In 2004, two studies in *S. cerevisiae* described for the first time that Cdk1 is essential for DSB repair pathway choice by promoting DNA-end resection in G2 phase (Aylon et al., 2004; Ira et al., 2004). These findings were later confirmed in human cells, showing that ssDNA-dependent activation of the ATR checkpoint pathway in response to DSBs is restricted to S/G2 and requires CDK activity (Jazayeri et al., 2006). Similarly, inhibition of CDK2 in mammalian cells was shown to impair HR and delay DSB signaling (Deans et al., 2006). Based on these key findings, it was proposed that DNA-end resection is governed by CDK-mediated phosphorylation (Figure 1) (Ira et al., 2004). However, it was only until the last few years that components of the resection machinery were identified as CDK substrates.

### MRX/MRN

Genetic studies in *S. cerevisiae* have long implicated the Mre11-Rad50-Xrs2 (MRX) complex in the initial processing of DSBs (Symington and Gautier, 2011). However, as MRX exhibits both endonuclease and 3′-5′ exonuclease activities *in vitro* (Paull, 2010), it still remains unclear how MRX catalyzes 5′-3′ nucleolytic degradation of DNA ends *in vivo*. New clues came from a recent study suggesting that DNA-end resection could occur with bidirectional polarity, as opposed to the unidirectional model shown in Figure 1. Accordingly, Mre11 endonuclease first creates a nick in the strand to be resected up to 300 nucleotides away from the DSB that, in a second step, serves as an entry point for resection by Mre11 3′-5′ exonuclease toward the DSB end and by Exo1 5′-3′ exonuclease away from the DSB (Garcia et al., 2011).

None of the MRX subunits have so far been reported as Cdk1 substrates. Moreover, an *mre11* mutant in which all six S/T-P motifs have been mutagenized did not exhibit any major phenotypes attributable to a resection defect. The same holds true for an *xrs2* mutant in which both CDK consensus motifs (S/T-P-x-K/R) were mutated (Ira et al., 2004). Notably, however, three additional S/T-P motifs in Xrs2 were found to be phosphorylated in a proteomic study, raising the possibility of it being indeed a Cdk1 substrate (Albuquerque et al., 2008). In human cells, akin to the situation in yeast, only the NBS1 subunit of the MRN complex was found to be phosphorylated in a cell-cycle-dependent manner (Figure 1; Olsen et al., 2010). Additionally, two groups reported that CDKs phosphorylate NBS1 at serine 432 in S phase (Falck et al., 2012; Wohlbold et al., 2012). Surprisingly, while Falck et al. concluded that NBS1-S432 phosphorylation promotes DNA-end resection, Wohlbold et al. reported normal resection in the absence of NBS1-S432 phosphorylation. Although it is rather difficult to reconcile these contradicting results, they have most likely emanated from the different NBS1-deficient cells used for complementation studies. Thus, it remains to be clarified whether Xrs2/NBS1 phosphorylation by CDKs is a conserved mechanism to promote DNA-end resection by MRX/N.

### Sae2/CTIP

*SAE2* (or *COM1*) was originally identified as being required to complete meiotic recombination in *S. cerevisiae* (McKee and Kleckner, 1997; Prinz et al., 1997). Subsequent genetic and biochemical studies in yeast and mammalian cells have shown that Sae2 and its human counterpart CtIP cooperates with the MRX/N nuclease to initiate resection of DSBs (Figure 1; Sartori et al., 2007; Symington and Gautier, 2011). There are three potential CDK phosphorylation sites in Sae2 and 12 in CtIP. Remarkably, phosphorylation of a single S/T-P motif in the C-terminus of both proteins (Sae2-S267/CtIP-T847) by CDK is required to promote resection (Huertas et al., 2008; Huertas and Jackson, 2009). Consistent with a role of Cdk1 in positively regulating Sae2 function, mutation of a RxL cyclin-binding motif present upstream of S267 caused comparable DNA damage hypersensitivity to that of *sae2-S267A* cells (Huertas et al., 2008). Moreover, in cells expressing a phospho-mimicking mutant (Sae2-S267E/CtIP-T847E), resection is permitted even in absence of Cdk1 activity; however, not to the same extent as in normal cells. Therefore, it was proposed that additional Cdk1 sites, on Sae2/CtIP itself or on other proteins, are required for optimal resection (Huertas, 2010). Despite the fact that the precise mechanism of how S267/T847 phosphorylation “activates” Sae2/CtIP is still unclear, it is of major importance for both meiotic and mitotic recombination (Manfrini et al., 2010; Nicolette et al., 2010).

Prior to the identification of CtIP-T847 as a CDK site, phosphorylation of S327 was shown to occur exclusively during S/G2 and to be a pre-requisite for CtIP-BRCA1 interaction (Yu and Chen, 2004; Yu et al., 2006). Furthermore, it was recently shown that CtIP-S327 phosphorylation is CDK2-dependent and facilitated by MRE11, which directly interacts with CDK2 and CtIP, thereby bringing CDK2 in proximity with its substrate (Buis et al., 2012). Although evidence for a direct role of CtIP-S327 phosphorylation in resection is still missing, the BRCA1-CtIP complex was recently reported to facilitate the removal of the 53BP1 effector protein RIF1 from DSBs in S/G2, thereby channeling DSB repair into HR (Escribano-Díaz et al., 2013). Moreover, it was recently reported that phosphorylation of a cluster of five additional S/T-P motifs located in the central region of CtIP is important for DNA-end resection (Wang et al., 2013). Mechanistically, phosphorylation of this cluster is needed for the association of CtIP with NBS1, which promotes DNA damage-induced CtIP phosphorylation by ATM (You et al., 2009; Wang et al., 2013). It is important to note, however, that Wang et al. did not directly address whether any of these clustered phosphosites in CtIP are indeed targeted by CDKs *in vivo*.

### Ku

When DSBs arise in the cell, Ku—a heterodimer composed of Ku70 and Ku80—is usually loaded onto duplex DNA ends. During the repair process, Ku serves as a docking site for many NHEJ proteins, including DNA-PKcs and DNA ligase IV, to rejoin the broken ends (Lieber, 2010). It has been shown that DNA-end resection and HR are constrained during G1 due to both efficient NHEJ and low CDK activity (Aylon et al., 2004; Jazayeri et al., 2006). Interestingly, in the absence of Ku, Cdk1 activity is dispensable for the initiation of resection by MRX-Sae2, but is still needed for long-range resection by Exo1 or Sgs1-Dna2 (Clerici et al., 2008). Therefore, Ku is thought to antagonize DNA-end resection and has to be removed from the ends in order to permit HR. These data also indicate that CDK activity promotes resection by restraining the recruitment of Ku to DSBs, raising the question whether Ku itself is a potential CDK substrate (Figure 1). However, removal of all putative Cdk1 phosphorylation sites on Ku70 and 3 out of 4 sites on Ku80 failed to elicit any DSB repair phenotype in *S. cerevisiae*, suggesting that the negative regulation of Ku by Cdk1 is most likely indirect (Zhang et al., 2009). Ku binding to DNA ends also attenuates resection and HR in mammalian cells (Shao et al., 2012; Tomimatsu et al., 2012). Furthermore, Ku70 was reported as a binding partner and substrate of CDK2-cyclin A, and Ku70-T455 was identified as a CDK target site by mass spectrometry (Müller-Tidow et al., 2004; Chi et al., 2008; Olsen et al., 2010); but whether or not Ku phosphorylation by CDKs has an impact on DNA-end resection has yet to be determined.

### Exo1

Exonuclease 1 (Exo1) belongs to the RAD2/XPG family of structure-specific 5′ nucleases and has been implicated in multiple genome maintenance pathways including DNA repair and telomere maintenance (Tran et al., 2004). Exo1 is dispensable for initial resection in yeast and human cells but acts in a separate pathway from Sgs1-Dna2/BLM-DNA2 to promote extensive 5′-3′ DSB resection (Figure 1; Symington and Gautier, 2011). Moreover, Exo1-dependent resection and its recruitment to DSBs depends on both MRX/N and Sae2/CtIP and is blocked by the presence of Ku (Eid et al., 2010; Sun et al., 2012; Tomimatsu et al., 2012). Although DNA damage-induced phosphorylation of Exo1 has been reported to attenuate its activity in both yeast and human cells (Morin et al., 2008; Bolderson et al., 2010), probably by controlling its stability (El-Shemerly et al., 2005), there is currently no published data available whether Exo1 is a CDK target. However, several S/T-P sites in human EXO1 were repeatedly found to be phosphorylated using mass spectrometry analyses (El-Shemerly et al., 2008; Chen et al., 2009; Shiromizu et al., 2013). Indeed, some of these sites are phosphorylated by CDKs in S/G2 phase, thereby stimulating DNA-end resection by EXO1 and promoting DSB repair by HR while at the same time suppressing NHEJ (S. Burma, personal communication).

### Sgs1-Dna2/BLM-DNA2

Sgs1 and its human ortholog BLM are members of the RecQ family of 3′-5′ DNA helicases and are involved in the suppression of crossovers by promoting the dissolution of Holliday junction intermediates (Bernstein et al., 2010). The role for Sgs1 in conjunction with the Dna2 nuclease in the generation of long stretches of ssDNA during HR was discovered because of its redundancy with Exo1 (Figure 1; Gravel et al., 2008; Mimitou and Symington, 2008; Zhu et al., 2008). Although there is currently no data available on CDK-mediated phosphorylation of Sgs1, BLM is phosphorylated at various S/T-P motifs by mitotic kinases including CDK1 (Beausoleil et al., 2004; Leng et al., 2006; Dephoure et al., 2008; Olsen et al., 2010). However, these modifications are more likely to be involved in the regulation of BLM's function in the separation of sister chromatids during mitosis rather than in DNA-end resection (Chan and Hickson, 2011). In contrast, Cdk1-mediated phosphorylation of *S. cerevisiae* Dna2 at T4, S17, and S237 stimulates its recruitment to DSBs and DNA-end resection (Chen et al., 2011). Consistent with the redundancy observed between Dna2- and Exo1-dependent resection pathways, *dna2-T4A/S17A/S237A* cells only resect DSBs in the presence of functional Exo1. Interestingly, T4 and S17 lie within a bipartite nuclear localization signal, suggesting a timely regulated nuclear import of Dna2 upon phosphorylation during G1/S transition (Kosugi et al., 2009). Remarkably, human DNA2 lacks the entire N-terminal region of yeast Dna2 including all three S/T-P sites, suggesting that CDK-mediated regulation of long-range resection in human cells differs from yeast.

### RPA

Replication protein A (RPA) is an evolutionarily conserved, heterotrimeric complex consisting of RPA1, RPA2, and RPA3. Owing to its high ssDNA binding affinity, RPA is required for most aspects of DNA metabolism including replication, repair and recombination (Oakley and Patrick, 2010). Following resection, RPA wraps around the generated 3′-ssDNA overhangs to protect the DNA against nuclease degradation and to prevent hairpin formation that would impede Rad51 filament assembly (Figure 1; Holloman, 2011). *In vitro* studies have also implicated RPA in promoting long-range resection through stimulation of both Exo1- and Sgs1-Dna2-dependent pathways (Cejka et al., 2010; Niu et al., 2010; Nimonkar et al., 2011; Cannavo et al., 2013). Furthermore, under DNA-damaging conditions, RPA-coated ssDNA serves to recruit the Mec1/ATR kinase, a critical event in checkpoint activation (Zou and Elledge, 2003). RPA2 contains a flexible N-terminal domain that is differentially phosphorylated at multiple residues during the cell cycle and in response to genotoxic stress. Two residues within this region, S23 and S29, are phosphorylated by CDK2-cyclin A and CDK1-cyclin B at the G1/S boundary and during mitosis, respectively (Figure 1); however, they are not conserved in yeast (Oakley and Patrick, 2010). In response to DSBs, ATR-mediated phosphorylation of RPA2-S33 induces phosphorylation of RPA2-S23/S29, and both act synergistically to stimulate phosphorylation of additional residues closer to the N-terminus by DNA-PK (Anantha et al., 2007; Liaw et al., 2011). Although DNA damage-induced RPA2 hyper-phosphorylation seems critical for Rad51 recruitment and HR in response to replication stress, it is not essential for HR as measured by an I-SceI-based reporter assay (Shi et al., 2010; Serrano et al., 2013). Moreover, dephosphorylation of RPA2 by the PP4 phosphatase complex has also been reported to facilitate HR (Lee et al., 2010). However, a direct role of CDK-mediated RPA phosphorylation in DNA-end resection has not yet been demonstrated.

### Chromatin binding and remodelling factors

DNA-end resection occurs in the context of chromatin, which constitutes a natural barrier to all kind of DNA transactions including DSB repair (Price and D'Andrea, 2013; Tsabar and Haber, 2013). Last year, three groups described a role of the *S. cerevisiae* chromatin-remodeling factor Fun30 (and its human counterpart SMARCAD1) in the repair of DSBs by HR (Chen et al., 2012; Costelloe et al., 2012; Eapen et al., 2012). Fun30/SMARCAD1 physically associates with DSB ends and, by weakening the histone-DNA interactions in nucleosomes, establishes a DNA conformation that facilitates both Sgs1- and Exo1-dependent resection. Furthermore, it was shown that Fun30 function in resection becomes less important in cells lacking the histone-bound Rad9 checkpoint protein, suggesting that Fun30 helps to overcome the inhibitory effect of Rad9 on DNA-end resection (Chen et al., 2012). Interestingly, both Fun30 and Rad9 were identified as Cdk1 substrates and reported to be phosphorylated at multiple S/T-P sites (Ubersax et al., 2003; Albuquerque et al., 2008). Moreover, loss of Rad9 has been reported to partially bypass the requirement for Cdk1 in resection (Lazzaro et al., 2008). This inhibitory mechanism is likely to be evolutionarily conserved as 53BP1, the mammalian ortholog of Rad9 (Wang et al., 2002), suppresses resection to promote NHEJ and immunoglobulin class switching (Bunting et al., 2010; Bothmer et al., 2011). Accordingly, multiple CDK consensus sites in SMARCAD1 and 53BP1 were repeatedly found to be phosphorylated (Beausoleil et al., 2004; Linding et al., 2007; Bennetzen et al., 2010; Olsen et al., 2010; Shiromizu et al., 2013). Further experiments are required to establish whether some of the CDK sites in Fun30/SMARCAD1 and Rad9/53BP1 play a role in the regulation of DNA-end resection and, thus, in DSB repair pathway choice.

## Concluding remarks

While the role of CDKs in regulating DNA-end resection is a given fact, we are only beginning to understand the mechanistic consequences of these phosphorylation events for individual repair factors, e.g., on protein-protein interactions, intracellular localization, or protein stability. Another important question to address in the future is how DNA-end resection is limited in order to generate confined tracts of ssDNA that are suitable for homology search by the Rad51 recombinase leading to productive HR. In other words, there must be additional regulatory mechanisms providing a switch between activation and inhibition of DNA-end resection to coordinate DSB repair pathways in a spatiotemporal manner.

Novel insights are provided by a recent study showing that PIN1, a phosphorylation-specific peptidyl-prolyl cis/trans isomerase, counteracts DNA-end resection in human cells (Steger et al., 2013). PIN1 was previously shown to isomerize phosphorylated S/T-P peptide bonds, thereby controlling the function of a subset of CDK substrates involved in diverse cellular processes (Liou et al., 2011). In a proteomic screen for PIN1 substrates, Steger et al. identified several prominent DSB repair proteins including BRCA1, 53BP1 and CtIP. Interestingly, PIN1-mediated isomerization of CtIP requires the phosphorylation of CtIP at two S/T-P sites: CtIP-pT315 (by CDK) serves as the major binding site for PIN1, whereas CtIP-pS276 (by an unknown proline-directed kinase) is isomerized by PIN1. Following isomerization, CtIP gets ubiquitylated and subsequently degraded by the proteasome. In this way, PIN1 is proposed to limit DNA-end resection, thereby possibly contributing to fine-tune the coordination of HR and NHEJ during S and G2 phases of the cell cycle (Figure 1; Karanam et al., 2012). So far, no direct connection has been made between PIN1 and the regulation of DSB repair in *S. cerevisiae*, studies of which are hampered by the fact that yeast PIN1 (Ess1) is essential for viability (Siepe and Jentsch, 2009). Future studies will have to determine whether phosphorylation-dependent regulation by PIN1 in concert with CDKs applies to other DSB repair proteins apart from CtIP and, thus, represents a general feature of the DDR.

### Conflict of interest statement

The authors declare that the research was conducted in the absence of any commercial or financial relationships that could be construed as a potential conflict of interest.
